# Assessment of compliance with US Public Health Service Clinical Practice Guideline for tobacco by primary care physicians

**DOI:** 10.1186/s12954-015-0044-3

**Published:** 2015-03-07

**Authors:** Judy Kruger, Alissa O’Halloran, Abby Rosenthal

**Affiliations:** Office on Smoking and Health, National Center for Chronic Disease Prevention and Health Promotion, Centers for Disease Control and Prevention, CDC4770 Buford Highway, Chamblee, Building 107, M/S, F-79, Atlanta, GA 30341-3717 USA; Contractor Support for NCCDPHP/NGIS, Office on Smoking and Health, National Center for Chronic Disease Prevention and Health Promotion, Centers for Disease Control and Prevention, Atlanta, GA 30341-3717 USA; Health Systems Consulting, Atlanta, GA 30341 USA

**Keywords:** Smoking cessation, USPHS Clinical Guideline, Tobacco, Primary care physicians, Health professionals

## Abstract

**Background:**

The US Public Health Service Clinical Practice Guideline *Treating Tobacco Use and Dependence: 2008 Update* established an expanded standard of care, calling on physicians to consistently identify their patients who use tobacco and treat them using counseling and medication.

**Findings:**

To assess compliance, we examined the extent to which physicians self-report following four of the five components of the 5A model: *Ask* about tobacco use, *Advise* patients who use tobacco to quit, *Assist* the patient in making a quit attempt, and *Arrange* for follow-up care. We used data from a Web-based panel survey administered to a convenience sample of 1,253 primary care providers (family/general practitioners, internists, and obstetrician/gynecologists). We found that 97.1% of the providers reported that they consistently *Asked* and documented tobacco use, while 98.6% reported that they consistently *Advised* their patients to quit using tobacco. Among the family/general practitioners and internists, 98.3% recommended “any” (medication, counseling, counseling and medication, telephone quitline) smoking cessation strategies (*Assist*). Among all providers, 48.0% reported that they consistently scheduled a follow-up visit (*Arrange*).

**Conclusions:**

This study revealed that most primary care physicians reported that they *Ask* their patients about tobacco use, *Advise* them to quit, and *Assist* them in making a quit attempt, but only half reported that they *Arrange* a follow-up visit. Tobacco use screening and intervention are among the most effective clinical preventive services; thus, efforts to educate, encourage, and support primary care physicians to provide evidence-based treatments to their patients should be continued.

## Introduction

Information on how physicians apply the 2008 US Public Health Service (USPHS) Clinical Practice Guideline recommendations on helping tobacco users quit can help facilitate the adoption of a brief intervention known as the 5A’s: *Ask*, *Advise*, *Assist*, *Arrange*, and *Assess* [1]. Most smokers need to make multiple quit attempts before they quit [2], and only 7% of smokers who attempt to quit without any cessation assistance are successful [3]; thus, primary care providers must address cessation repeatedly with their patients who use tobacco [4].

Findings from the International Agency for Research on Cancer concluded that while population-level interventions have proven far more effective than individual-based interventions, brief advice from physicians to quit smoking is effective in comparison to other individually focused interventions [5]. The USPHS Guideline concluded that the provision of both medication support and counseling by physicians can increase the probability of quitting by 30% compared to medication alone [1]. Since about 70% of adult smokers visit a physician each year, physicians have many opportunities to help their patients who use tobacco by using counseling and medication [6].

Medicare, many state programs, and, more recently, Medicaid have expanded coverage of cessation treatments [7]. Provisions in the 2010 Affordable Care Act have expanded private and Medicaid cessation coverage. As of September 23, 2010, non-grandfathered private plans were required to cover, without cost-sharing, preventive services that have an “A” or “B” recommendation from the US Preventive Services Task Force, including tobacco cessation interventions [8]. On May 2, 2014, the US Departments of Health and Human Services, Labor, and the Treasury jointly issued subregulatory guidance on implementing this requirement [9]. The number of physicians who implement the USPHS Guideline may increase as knowledge of the requirement expands [10]. Monitoring implementation of the key tobacco cessation treatment recommendations is important, given the expansion of coverage for cessation counseling and medication and because of the potential increase in the number of smokers who visit a physician annually [11].

Multiple studies have assessed the prevalence of patient-reported receipt of various components of the 5A model; however, there is little physician-reported evidence on the extent to which they provide these services to patients [12,13]. We analyzed data from the 2011 DocStyles survey to determine the percentage of primary care providers who report providing four of the five components of the 5A’s: *Ask*, *Advise*, *Assist*, and *Arrange*; the dataset did not allow examination of the *Assess* component. We also estimated the percentage of providers who reported recommending both counseling and medication.

## Methods

Data were obtained from the 2011 DocStyles, a Web-based panel survey administered to a convenience sample of health care providers. A random sample of eligible health providers was selected from the Epocrates Honors Panel, an opt-in, verified panel of over 190,000 medical practitioners [14]. This sample was drawn to match the American Medical Association’s (AMA) master data file proportions for age, gender, and region. Electronic invitations included a link to the Web-based survey; providers were eligible to participate if they were practicing in the United States, were actively seeing patients, and had been practicing for at least 3 years. Other studies using DocStyles have reported details of the survey design and data collection procedures [15,16].

We invited 4,097 health care providers to participate during July to August 2011; of these, 2,204 (62.8%) completed the entire survey. Because we wanted to assess compliance with the USPHS Guideline, we excluded those who were not primary care providers; the final sample (*N* = 1,253) consisted of family/general practitioners, internists, and obstetrician/gynecologists. Compared to the AMA master file, respondents in our sample were disproportionately men and slightly younger in age, though there were no differences by US region (data not shown).

### Measures

#### USPHS Clinical Practice Guideline

All primary care providers were asked four questions related to their tobacco treatment behaviors. Providers were asked if they consistently *Asked* their patients if they smoke, *Advised* them to quit, *Assisted* with quitting, and *Arranged* follow-up. There were no skip patterns, so all primary care providers were asked each question and, thus, a respondent could have answered No to the *Ask* question and Yes to the *Advise* question. Respondents could select multiple options for the *Assist* question.

#### Demographic and physician practice characteristics

Demographic characteristics included sex, age, and race/ethnicity. Provider practice characteristics included primary care provider type (family/general practice, internal medicine, or obstetrics/gynecology), teaching privileges (yes or no), type of practice (individual private practice, group private practice, or hospital/clinic practice), years in practice, number of physicians in practice, and estimated number of patients seen per week.

### Analysis

Data were analyzed using SAS-Callable SUDAAN 10 (RTI International, Research Triangle Park, NC). Descriptive statistics were calculated to determine the number of physicians who self-reported providing various components of the 5A model to their patients. Point estimates and 95% confidence intervals were calculated to examine specific *Assist* outcomes, including 1) providing coaching or counseling, 2) prescribing or recommending a tobacco cessation medication, 3) asking patients to call a tobacco quitline, 4) any of these, or 5) the combination of counseling and medication (the two core recommendations of the USPHS Guideline).

## Results

Of these primary care physicians, 46.6% were family/general practitioners, 33.4% were internists, and 20.0% were obstetricians/gynecologists (Table 1). Most respondents were men and were 36–45 years of age, non-Hispanic white, and in a hospital-based clinic or individual practice. Approximately one third of the respondents were in practice 11–20 years, were in a practice with 3–5 other physicians, and saw about 76–100 patients per week.

**Table 1:** **Prevalence of demographic and practice characteristics of primary care provider sample—DocStyles survey, 2011**

**Characteristics**	***N*** ^**a**^	**%**	**95% CI**
Sex			
Men	883	70.5	67.9, 73.0
Women	370	29.5	27.0, 32.1
Age			
26–35	147	11.7	9.9, 13.5
36–45	541	43.2	40.4, 45.9
46–55	340	27.1	24.7, 29.6
≥56	225	18.0	15.8, 20.1
Race/ethnicity			
Non-Hispanic white	802	64.0	61.3, 66.7
Non-Hispanic black	56	4.5	3.3, 5.6
Hispanic	63	5.0	3.8, 6.2
Non-Hispanic other	332	26.5	24.1, 28.9
Primary care provider type			
Family/general practitioners	584	46.6	43.8, 49.4
Internists	418	33.4	30.7, 36.0
Obstetrician/gynecologists	251	20.0	17.8, 22.2
Teaching privileges			
Yes	637	50.8	48.1, 53.6
No	616	49.2	46.4, 51.9
Type of practice			
Individual	221	17.6	15.5, 19.7
Group	812	64.8	62.2, 67.4
Hospital/clinic	220	17.6	15.5, 19.7
Years in practice			
≤5	109	8.7	7.1, 10.3
6–10	353	28.2	25.7, 30.7
11–20	472	37.7	35.0, 40.4
≥21	319	25.5	23.0, 27.9
Number of physicians in practice			
1–2	316	25.2	22.8, 27.6
3–5	345	27.5	25.1, 30.0
6–10	247	19.7	17.5, 21.9
11–25	181	14.4	12.5, 16.4
≥26	164	13.1	11.2, 15.0
Number of patients per week			
1–75	242	19.3	17.1, 21.5
76–100	479	38.2	35.5, 40.9
101–150	394	31.4	28.9, 34.0
≥151	138	11.0	9.3, 12.7
Total	1,253	--	--

*CI* confidence interval.

^a^Total unweighted number of respondents.

Among the providers in this sample, 97.1% reported that they *Asked* about smoking, 98.6% *Advised* tobacco users to quit using tobacco products, 98.3% *Assisted* tobacco users to quit, and 48.0% *Arranged* a follow-up visit (Table 2). The proportion of physicians who reported arranging for follow-up was higher among physicians aged 26–35 years of age than all other age groups, family practitioners and internists than OB/GYNs, physicians with teaching privileges than those without, those with less years of practice, and those who saw more patients per week.

**Table 2:** **Prevalence of primary care providers’ advice practices toward quitting** ^**a**^ **—DocStyles survey, 2011**

**Characteristic**	**Ask**	**Advise**	**Assist**					**Arrange**
	**Asked about smoking**	**Advised to quit using tobacco** ^**b**^	**Prescribed or recommended medication**	**Recommended coaching or counseling**	**Recommended coaching or counseling and medication**	**Recommended using telephone quitlines**	**Offered any assistance**	**Scheduled a follow-up**
	**% (95% CI)**	**% (95% CI)**	**% (95% CI)**	**% (95% CI)**	**% (95% CI)**	**% (95% CI)**	**% (95% CI)**	**% (95% CI)**
Sex								
Men	97.3 (96.0, 98.2)	98.8 (97.8, 99.3)	91.6 (89.3, 93.5)	87.7 (85.5, 89.8)	83.3 (80.5, 86.0)	52.1 (48.8, 55.4)	98.1 (96.9, 98.8)	48.4 (45.1, 51.7)
Women	96.8 (94.4, 98.1)	98.1 (96.1, 99.1)	95.3 (92.2, 97.2)	88.9 (85.7, 92.1)	87.5 (83.8, 91.3)	59.2 (54.2, 64.2)	98.9 (97.2, 99.6)	47.0 (41.9, 52.1)
Age								
26–35	98.6 (94.7, 99.7)	98.6 (94.7, 99.7)	91.7 (85.6, 95.3)	91.8 (86.2, 95.3)	84.8 (78.7, 91.0)	69.4 (61.9, 76.8)	99.3 (95.3, 99.9)	61.2 (53.3, 69.1)
36–45	98.0 (96.4, 98.9)	98.3 (96.8, 99.1)	92.5 (89.6, 94.6)	88.7 (86.1, 91.4)	84.5 (81.2, 87.9)	52.3 (48.1, 56.5)	98.3 (96.8, 99.1)	48.8 (44.6, 53.0)
46–55	95.9 (93.2, 97.5)	99.1 (97.3, 99.7)	92.8 (89.0, 95.4)	89.1 (85.8, 92.4)	85.7 (81.4, 89.9)	53.8 (48.5, 59.1)	99.7 (97.9, 100.0)	43.5 (38.3, 48.8)
≥56	96.0 (92.5, 97.9)	98.2 (95.4, 99.3)	93.9 (89.1, 96.7)	82.2 (77.2, 87.2)	82.4 (76.6, 88.2)	49.3 (42.8, 55.9)	95.6 (91.9, 97.6)	44.0 (37.5, 50.5)
Race/ethnicity								
Non-Hispanic white	97.0 (95.6, 98.0)	98.5 (97.4, 99.1)	94.4 (92.3, 96.0)	88.7 (86.5, 90.8)	87.2 (84.6, 89.9)	53.5 (50.0, 56.9)	98.5 (97.4, 99.1)	44.8 (41.3, 48.2)
Non-Hispanic black	100.0 (100.0, 100.0)	96.4 (86.8, 99.1)	91.5 (79.4, 96.8)^c^	83.9 (74.3, 93.6)^c^	78.7 (67.0, 90.4)^c^	55.4 (42.3, 68.4)^c^	96.4 (86.8, 99.1)^c^	58.9 (46.0, 71.8)^c^
Hispanic	96.8 (88.2, 99.2)	98.4 (89.6, 99.8)	93.6 (82.0, 97.9)^c^	92.1 (82.3, 96.7)^c^	87.2 (77.7, 96.8)^c^	42.9 (30.6, 55.1) ^c^	98.4 (89.6, 99.8)^c^	65.1 (53.3, 76.9)^c^
Non-Hispanic other	97.0 (94.5, 98.4)	99.1 (97.2, 99.7)	89.3 (85.7, 92.8)	86.4 (82.8, 90.1)	79.5 (74.9, 84.1)	57.8 (52.5, 63.1)	98.2 (96.0, 99.2)	50.6 (45.2, 56.0)
Primary care provider type								
Family/general practitioners	96.1 (94.1, 97.4)	98.6 (97.3, 99.3)	94.0 (91.8, 95.7)	90.4 (87.7, 92.5)	86.0 (83.1, 88.8)	61.1 (57.2, 65.1)	98.8 (97.5, 99.4)	53.1 (49.0, 57.1)
Internists	98.1 (96.2, 99.0)	98.8 (97.2, 99.5)	90.9 (87.7, 93.3)^d^	90.0 (86.7, 92.5)	82.5 (78.9, 86.2)^d^	50.5 (45.7, 55.3)	99.0 (97.5, 99.6)	56.0 (51.2, 60.7)
Obstetrician/gynecologists	98.0 (95.3, 99.2)	98.0 (95.3, 99.2)		79.3 (74.3, 84.3)		44.2 (38.1, 50.4)	96.0 (92.8, 97.8)	22.7 (17.5, 27.9)
Teaching privileges								
Yes	97.2 (95.6, 98.2)	98.3 (96.9, 99.0)	92.9 (90.3, 94.9)	86.3 (83.7, 89.0)	84.0 (80.8, 87.3)	54.2 (50.3, 58.0)	98.3 (96.9, 99.0)	51.3 (47.5, 55.2)
No	97.1 (95.4, 98.2)	98.9 (97.6, 99.5)	92.5 (89.9, 94.5)	89.8 (87.4, 92.2)	85.0 (81.9, 88.1)	54.2 (50.3, 58.2)	98.4 (97.0, 99.1)	44.5 (40.6, 48.4)
Type of practice								
Individual	97.7 (94.7, 99.1)	99.1 (96.5, 99.8)	91.4 (86.3, 94.6)	86.4 (81.9, 90.9)	80.5 (74.8, 86.2)	48.0 (41.4, 54.6)	96.8 (93.5, 98.5)	50.7 (44.1, 57.3)
Group	96.9 (95.5, 97.9)	98.6 (97.6, 99.2)	94.1 (91.9, 95.7)	88.8 (86.6, 91.0)	86.4 (83.7, 89.1)	54.7 (51.3, 58.1)	98.9 (97.9, 99.4)	46.6 (43.1, 50.0)
Hospital/clinic	97.3 (94.1, 98.8)	97.7 (94.7, 99.1)	89.5 (85.2, 93.9)	86.8 (82.3, 91.3)	82.2 (76.8, 87.6)	58.6 (52.1, 65.1)	97.7 (94.7, 99.1)	50.5 (43.8, 57.1)
Years in practice								
≤5	98.2 (93.0, 99.5)	99.1 (93.8, 99.9)	92.2 (85.1, 96.0)	89.9 (84.3, 95.6)	83.3 (76.1, 90.6)	61.5 (52.3, 70.6)	100.0 (100.0, 100.0)	61.5 (52.3, 70.6)
6–10	96.9 (94.5, 98.3)	98.3 (96.3, 99.2)	91.4 (87.7, 94.1)	90.4 (86.8, 93.0)	82.8 (78.5, 87.0)	57.2 (52.1, 62.4)	98.6 (96.6, 99.4)	50.7 (45.5, 55.9)
11–20	98.1 (96.4, 99.0)	98.5 (96.9, 99.3)	94.2 (91.2, 96.2)	89.0 (86.2, 91.8)	88.1 (84.7, 91.4)	54.4 (50.0, 58.9)	98.5 (96.9, 99.3)	47.9 (43.4, 52.4)
≥21	95.6 (92.7, 97.4)	98.7 (96.7, 99.5)	92.4 (88.3, 95.2)	83.4 (79.3, 87.5)	81.9 (77.0, 86.8)	48.0 (42.5, 53.4)	97.2 (94.7, 98.5)	40.4 (35.1, 45.8)
Number of physicians in practice								
1–2	97.8 (95.4, 98.9)	99.7 (97.8, 100.0)	93.1 (89.2, 95.6)	87.0 (83.3, 90.7)	82.6 (78.0, 87.2)	47.5 (42.0, 53.0)	97.8 (95.4, 98.9)	46.5 (41.0, 52.0)
3–5	96.5 (94.0, 98.0)	97.7 (95.4, 98.8)	91.9 (88.0, 94.6)	87.0 (83.4, 90.5)	83.4 (79.0, 87.8)	51.6 (46.3, 56.9)	98.8 (97.0, 99.6)	47.8 (42.6, 53.1)
6–10	97.2 (94.2, 98.6)	98.0 (95.2, 99.2)	93.6 (89.0, 96.3)	87.4 (83.3, 91.6)	84.5 (79.3, 89.7)	59.1 (53.0, 65.2)	98.8 (96.3, 99.6)	47.0 (40.7, 53.2)
11–25	97.2 (93.5, 98.8)	98.3 (95.0, 99.5)	90.8 (85.1, 94.5)	91.2 (86.1, 94.5)	86.3 (80.8, 91.7)	61.9 (54.8, 69.0)	97.8 (94.3, 99.2)	51.4 (44.1, 58.7)
≥26	97.0 (92.9, 98.7)	99.4 (95.8, 99.9)	94.7 (89.3, 97.5)	89.6 (85.0, 94.3)	88.6 (83.2, 94.1)	56.7 (49.1, 64.3)	98.2 (94.5, 99.4)	48.8 (41.1, 56.4)
Number of patients per week								
1–75	96.7 (93.5, 98.3)	99.2 (96.8, 99.8)	87.4 (82.7, 92.0)	87.6 (83.5, 91.8)	80.8 (75.3, 86.3)	55.4 (49.1, 61.6)	97.1 (94.1, 98.6)	40.9 (34.7, 47.1)
76–100	97.3 (95.4, 98.4)	98.3 (96.7, 99.2)	93.4 (90.5, 95.5)	89.4 (86.6, 92.1)	86.0 (82.6, 89.4)	58.5 (54.0, 62.9)	98.5 (97.0, 99.3)	48.2 (43.7, 52.7)
101–150	97.5 (95.3, 98.6)	98.7 (97.0, 99.5)	96.4 (93.6, 98.0)	87.3 (84.0, 90.6)	87.5 (83.8, 91.2)	49.5 (44.6, 54.4)	98.5 (96.7, 99.3)	47.0 (42.0, 51.9)
≥151	96.4 (91.6, 98.5)	97.8 (93.5, 99.3)	89.7 (84.0, 95.5)	86.2 (80.5, 92.0)	77.6 (69.7, 85.5)	50.7 (42.4, 59.1)	99.3 (95.0, 99.9)	62.3 (54.2, 70.4)
Total	97.1 (96.0, 97.9)	98.6 (97.7, 99.1)	92.7 (90.9, 94.2)	88.0 (86.2, 89.8)	84.5 (82.3, 86.8)	54.2 (51.4, 56.9)	98.3 (97.4, 98.9)	48.0 (45.2, 50.7)
*N*	1,217	1,235	929	1,103	847	679	1,232	601

Notes: Physicians were first asked the *Advise* question: “For tobacco users who visited you over the last year, did you consistently recommend they quit using tobacco?” They were next asked the *Ask* question: “For patients who visited you over the last year, did you consistently ask and document whether they use tobacco?” The third question was for the *Assist* component: “For tobacco users who visited you over the last year, did you consistently try to help them quit tobacco by doing any of the following…”; respondents could select all that applied from the possible options: 1) providing coaching or counseling, 2) prescribing or recommending a tobacco cessation medication, or 3) asking them to call a tobacco quitline. The final question asked of primary care providers was for the *Arrange* component: “For tobacco users who visited you over the last year, did you consistently schedule a follow-up visit to help them quit tobacco?”.

*CI* confidence interval.

^a^Questions asked of tobacco users who visited health care provider over the last year.

^b^The sample size for *Advise* is greater than that for *Ask* because it was the first question asked of respondents.

^c^Interpretation may be limited as the sample size was <50.

^d^We excluded obstetrician/gynecologists from the medication portion of the analysis because medication recommendations are not uniform across all populations (e.g., infants, children, and pregnant women). Calculations for prescribed or recommended medication, and recommended counseling and medication were obtained from 1,002 obstetrician/gynecologists.

Among the family/general practitioners and internists who *Assisted* by providing interventions to tobacco users, 92.7% prescribed or recommended a medication, 88.0% recommended counseling, 84.5% recommended both counseling and medication, and 54.2% recommended using telephone quitlines.

## Discussion

Our findings reveal that nearly all primary care providers reported that they consistently *Asked* their patients about tobacco use, *Advised* that their tobacco using patients quit, and *Assisted* tobacco using patients with “any” smoking cessation strategy. Fewer providers *Arranged* a follow-up visit with their patients to address tobacco use. A previous study found slightly lower estimates for provider self-reported delivery of *Ask* (95%), *Advise* (95%), *Assess* (91%), *Assist* (87%), and *Arrange* (17%); these variations could be due to differences in survey methodology, the questions used on the questionnaires, or subgroup distribution [12].

Approximately half (48%) of the primary care providers reported that they consistently *Arranged* a follow-up visit. Although this number was lower than that of the other 5A indicators reported here, the question only asked about one specific way of fulfilling the *Arrange* requirement: scheduling a follow-up visit and patients not interested in quitting may not be candidates for follow-up visits that are required by the USPHS Guideline. Our study found that disparities were observed across provider characteristics; primary care providers who self-reported that they scheduled follow-up contact related to a quit attempt were more likely to be younger physicians, family practitioners and internists, those with teaching privileges, those ≤5 years in practice, and those who reported seeing ≥151 patients per week. Offering follow-up assistance to smokers who attempt to quit maximizes the impact of these interventions on cessation; however, it is important to acknowledge that to *Arrange* follow-up takes more time than merely to *Ask* about smoking status and may require coordinated efforts from health care professionals in each provider’s office. Although not assessed as part of this study, telephone quitlines often provide a mechanism for follow-up that can optimize population coverage and health services used, promote community-based interventions, and develop partnerships with health care systems to support cessation and treatment [17]. Increasing primary care providers’ training on how to overcome barriers to implementation is warranted.

The *Assist* step also is important. Our study found a high prevalence of self-reported *Assist* recommendations (98.3% recommended medication, counseling, or telephone quitlines) compared to Conroy and colleagues’ findings that 79% of providers recommended any of these strategies [12]. Because there are disparate elements of the *Assist* step, it often needs to be tailored to each patient. To help maintain adherence to the *Assist* component, providers can develop office systems to bring screening and cessation interventions into routine practice [1,18] and communicate with patients using motivational interviewing techniques (e.g., express empathy, avoid arguing, support self-efficacy) [1,19]. The literature suggests that physicians can motivate patients who are not willing to make a quit attempt through enhanced communication skills [20-24].

The USPHS Guideline suggests that physicians recommend the use of counseling and medication as a combined intervention [1]. We found that 84.5% of family/general practitioners and internists recommended this combination to tobacco users. To our knowledge, this is the first study to evaluate primary care providers’ self-reported compliance with the guideline to *Assist* patients who use tobacco to quit. Because the combination of counseling and medication is more effective for smoking cessation than either counseling or medication alone, family/general practitioners and internists are encouraged to provide patients with both brief counseling and FDA-approved pharmacotherapies. Since these treatments are effective and now are available, every patient who uses tobacco should be offered, at least, brief cessation counseling, medication, and referrals [21,25].

These findings should be verified to confirm that physicians are consistently identifying and treating their patients who use tobacco at the high self-report rates found in this study, and that they are using both counseling and medication. This could be done via post-visit patient surveys, chart audits, and direct or recorded observation.

There are several important limitations of these data. First, though DocStyles is an opt-in Web-based survey designed to match specialty breakdown of the AMA membership, findings may not be representative of all primary care providers in the United States. Second, questions relied on recall of the previous 12 months, and it is possible that providers’ recall may not have accurately captured their behavior. Third, aspects of the 5A model questions may have caused discrepancies: 1) the dichotomous Yes/No response to questions included the word “consistently,” 2) the *Assess* component of the 5A model was not included in this study, and 3) the *Arrange* component only asked about follow-up visits and implied all tobacco users should always have such visits, which may have introduced response bias. Fourth, the survey did not address the full spectrum of the USPHS Guideline, which may limit how providers interpreted their provision of interventions. Fifth, survey responses were self-reported, which could lead to reporting bias; studies suggest that physicians may overestimate the extent to which they provide various components of the 5A model to their patients [12].

These findings highlight high levels of self-reported compliance with most components of the 5A model. Depending on how the *Arrange* question was interpreted by respondents, the 48% compliance response may suggest a need to increase efforts toward follow-up. Since the expanded standard of care using the evidence-based treatment recommended in the USPHS Guideline includes the combination of counseling and medication, efforts to educate, encourage, and support primary care physicians to provide these treatments should be continued.
